# Supplementary material for: Determinants of Consumers’ Acceptance and Adoption of Novel Food in View of More Resilient and Sustainable Food Systems in the EU: A Systematic Literature Review
Source: Foods. 2024 May 15;13(10):1534. doi: 10.3390/foods13101534 (PMC11120339; doi:10.3390/foods13101534)
Supplement: Supplementary file 1 [file foods-13-01534-s001.zip › Supplementary Table S1.pdf]

Table S1. Characteristics of the studies on aquatic products (M=Males; F=Females).

Authors	Year	Country	Participants' number and characteristics	Type of approach	Assessed variables	Type of product
Custódio et al. [52]	2021	Portugal	268 (M 42.5%; F 57.5%) Age: ≥ 18 years	Quantitative (in-person interviews)	Food related lifestyles, familiarity, willingness to pay	Halophyte-based products
		**Main Outcomes** Adventurous consumers and women show greater willingness to pay; strategies to improve familiarity and effective communication (about health effects and cooking possibilities) may help to increase acceptance				
Embling et al. [53]	2022	UK	476 (M 31.5%; F 68.3%) Age: ≥ 18 years	Quantitative (online questionnaire)	Beliefs (for 10 parameters - taste, edibility, healthiness, caloric content, naturalness, degree of processing, expensiveness, ethics, sustainability, and familiarity), liking, willingness to try, willingness to pay, readiness to adopt as meat alternative	Seaweeds-based food products
		**Main Outcomes** Taste/ edibility and familiarity were highlighted as strong drivers of acceptability, with taste/ edibility in particular identified as an attribute that could further enhance acceptance in consumers, and potentially mitigate the effects of food neophobia as a barrier to acceptance				
García-Segovia et al. [63]	2020	Spain	85 (M 72.9%; F 27.1%) Age: 25-69 years	Quantitative (in-person questionnaires)	Sensory evaluation (Check-All-That-Apply (CATA); Liking; Food neophobia	Microalgae based breadstick
		**Main outcomes** Sensory characteristics were reported as important aspects for acceptance of these products (crunchiness and saltiness are positive attributes, off-flavour is a negative attribute); healthiness perception is a driver of acceptance, whereas neophobia is a barrier				
Geertsen et al. [54]	2016	Denmark	200 (M 23%; F 77%) Age: ≥ 18 years	Quantitative (questionnaires and sensory evaluation)	Food neophobia; hedonic and sensory testing (CATA and liking); attitudes towards this type of product	Sea-buckthorn-based beverage
		**Main outcomes**				

		Familiarity was a barrier for these products; the main drivers of acceptance were sensory characteristics; neophobia did not have significant effect on acceptance				
Grahl et al. [55]	2020	Multi-country (Germany, Netherlands and France)	420 (M 47%; F 53%)<br>Age: 18-65 years	Quantitative (questionnaires and sensory evaluation)	Sensory testing (hedonics), food neophobia, familiarity, consumption behaviour	Filled pasta with spirulina-soy-extrudate
		**Main outcomes**<br>Flavour is a main barrier of acceptance for spirulina (masking taste appears promising); familiarity and neophilia promotes acceptance of these products. Comparing countries, France was more reluctant in accepting, due to different cultural gastronomic (sensory) background (reinforcing familiarity as driver)				
Lucas et al. [57]	2019	France	495 (M 48.4%; F 51.6%)<br>Age: > 15 years	Quantitative (in-person interviews)	Food habits (meat and fish consumption, frequency and type of seafood consumed, cooking habits, and curiosity); seaweeds consumers were asked about their perception (taste, cooking habits, etc.) and non-consumers were asked about the reasons to not consume; seaweeds knowledge and suggestion about solutions to simplify seaweed use; effect of label (label preference) on acceptance	Seaweeds
	**Main outcomes**<br>Curiosity about food, being used to cook seafood; not consuming meat daily, and possessing seaweed knowledge are drivers to accept/consume seaweeds; information about healthiness and sustainability, in labels, appeared as a driver for higher acceptance					
Michel et al. [64]	2021	Multi-country (Germany, France, UK)	1734 (M 51.7%; F 48.3%)<br>Age: 20-69 years	Quantitative (online questionnaires)	Meat products (hamburger) consumption; perception of expected taste; healthiness and environmental friendliness; meat commitment; food neophobia	Burger containing algae protein
		**Main outcomes**				

	Perception of healthiness and environmental friendliness was reported as a driver for acceptance; taste and meat commitment, as well as neophobia degree and negative attitudes towards vegetarian and vegan style are barriers. Country, age, and sex did not have significant influence on the expectation for the algae burger					
Moons et al. [66]	2018	Belgium	1325 (M 59%; F 41%)<br>Age: ≥ 18 years	Qualitative (through workshop); Quantitative (questionnaire)	Motivations to adopt spirulina-based products (qualitative studies); psychological parameters (model constructs), health consciousness, food involvement, food neophobia, willingness to compromise on taste, environmental concern; adoption intention towards spirulina-containing food products	Spirulina enhanced foods
	**Main outcomes**<br>Intention to adopt Spirulina-enhanced food was significantly higher for sport individuals, vegetarians and foodies than for life enjoyers; health consciousness was a driver for acceptance, as well as the willingness to compromise on taste; neophobia was a barrier for acceptance. Only in case of vegetarian, women were more prone to accept spirulina-based products					
Onwezen et al. [67]	2022	The Netherlands	Cross-sectional sample 4641 (M 53.2%; F 46.8%)<br>Longitudinal sample 500 (M 47.4%; F 52.6%)<br>Age: ≥ 18 years	Quantitative (online questionnaires)	Personal norms (health and environment); Food neophobia; Domain specific innovativeness; Food choice motives; Emotions; Intention to eat; Self-reported consumption	Seaweed
	**Main outcomes**<br>Positive emotions are highly relevant drivers for the intention to consume alternative proteins; Food neophobia and domain-specific innovation are determinants of self-reported consumption					
Torri et al. [60]	2020	Italy	1445 (M 46.4%; F 53.6%)<br>Age: 18-80 years	Quantitative (online questionnaires)	Food habits; personality related traits (food neophobia and sensitivity to disgust); attitude to jellyfish as an animal, attitude to jellyfish as food	Jellyfish

	**Main outcomes**					
	Food neophobia and sensitivity to disgust were main barriers for the acceptability of jellyfish, whereas younger age, familiarity with the sea environment, higher education level and openness to experiences as travelers were drivers					
Weinrich and Gassler [65]	2021	Germany	1175 (M 50.5%; F 49.4%)<br>Age: ≥ 16 years	Quantitative (online questionnaire)	Price sensitivity	Micro-algae based meat substitutes
	**Main outcomes**					
	Price sensitivity is a barrier					
