# Supplementary material for: Determinants of Consumers’ Acceptance and Adoption of Novel Food in View of More Resilient and Sustainable Food Systems in the EU: A Systematic Literature Review
Source: Foods. 2024 May 15;13(10):1534. doi: 10.3390/foods13101534 (PMC11120339; doi:10.3390/foods13101534)
Supplement: Supplementary file 1 [file foods-13-01534-s001.zip › Supplementary Table S2.pdf]

Table S2. Characteristics of the studies on insects (M=Males; F=Females).

Authors	Year	Country	Participants' number and characteristics	Type of approach	Assessed variables	Type of product
Arena et al. [85]	2020	Italy	210 (M 35.2% F 64.8%) Age range: 31-60 years	Quantitative (online questionnaire)	Previous experience of tasting insects or their derivatives; Eating habits; Food values; Food Neophobia; Expected Liking; Evoked sensations	Mix of edible insects, chocolate-coated grasshopper and scorpions, crickets, and silkworm flour protein bars (different levels of visibility of insects)
**Main outcomes** Information about edible insects led to an increase in food neophobia. Expected liking and beliefs about food values are not affected by the degree of information provided. Visibility of edible insects negatively affect the expected liking. As the edible insects become invisible in the product images, the feeling of disgust decreases, and curiosity increases as well as appetite. Healthiness, environmental impact, and naturalness are positive drivers in consuming insect-based foods whereas origin and appearance are the least recognized food values in insect-based foods						
Caparros Megido et al. [77]	2016	Belgium	79 (M 44% F56%) Age range: 18-25 years	Quantitative (questionnaire + sensory evaluation)	Previous experience with entomophagy; knowledge, perception of entomophagy; Liking data	Different burgers (beef burger, lentil burger, mealworm/beef burger (45% ground beef; 50% insects) mealworm/lentil burger (45% green lentils; 50% insects)
**Main outcome** The main preconception about entomophagy was curiosity. Beef burger was best liked, it followed in the order mealworm/beef burger, mealworm/lentil burger, and lentil burger. Higher liking of insect-based burger among men and for people with previous entomophagy experience or knowledge. About 4% of participants positively changed the perception of insect consumption thanks to overall liking and the taste of the burger						
Chow et al. [78]	2021	Denmark	238 (included data 148) (Gender % not reported) Age range: 11-13 years	Quantitative (questionnaire)	Neophobia; Cooking activities; Hedonic assessment (after tasting)	Insect based oatmeal balls (two types of insects; Grasshopper, larger and more visible; Mealworm, smaller and less visible)
**Main outcomes** In 10.5% of evaluations children were not willing to taste the insect oatmeal balls. Outcomes gave no evidence that children's willingness to taste and their hedonic responses to insect oatmeal balls were affected by their involvement in cooking activity. On the contrary, insect type, tasting order and children's food neophobia had significant influences on food acceptance. One unit increase in the neophobic direction of the Food Neophobia Taste Tool was estimated to decrease hedonic rating by 0.1 on the 7-point scale. First tasting of the food received a higher rating than the second time and mealworms oatmeal balls were more liked than the grasshopper version						
Cicatiello et al. [73]	2020	Italy	62 (M 47% F 53%) Age range: 18-35 years	Quantitative (questionnaire + sensory evaluation)	Food neophobia; General Neophobia; Personal attitude towards entomophagy; Food preferences and consumption habits;	Insect-based snacks (chocolate bar with insect flour, whole cricket,

					Liking after tasting of the appearance, odour, flavour, texture, and Overall liking; Overall evaluation of tasting experience	chips containing insect flour, caramel worms)

Main outcomes
The level of food neophobia among participants was found to be quite low. The participants referred low liking towards unusual foods such as offal and snails, whilst eating raw fish was much more common and appreciated. In response to whether they had prior experience with entomophagy, 19.5% of the participants declared that they had tasted insect-based products before. Among the four insect-based products, the highest overall liking was recorded for the bars, followed by crickets, and chips. Worms were scored the lowest. Liking was not affected by snacks containing whole, visible insects or by the main taste of the snack (sweet versus savoury), although a negative trend was seen regarding whole insects. Participant age demonstrated a negative effect, suggesting that younger participants had a higher liking of insect-based foods, although this did not reach significance

Çınar et al. [79]	2021	The Netherlands	Study 3: 160 (M 23% F 77%)<br>Study 4: 161 (M 20% F 80%)<br>Age range:<br>Study 3: 18–31 years<br>Study 4: 17–32 years	Quantitative (questionnaires + voluntaries sensory tasting	Food neophobia scale; Traditional Masculinity-Femininity Scale; Openness to Experience, Empathy Towards Animals Scale; Germ Aversion subscale of Perceived Vulnerability to Disease Scale; Pathogen Disgust subscale of the Three Domains of Disgust Scale.<br>To taste or not; for the ones who tasted: ratings of taste and texture; willingness to buy; Willingness to pay	Chocolate-orange flavored Bugbar, a commercially available snack bar that contains 5%

Main outcomes
Women scored higher on meat neophobia than men, but the sexes did not differ on plant neophobia. Most of the participants (78.6%) ate at least part of the Bugbar. Using regression analysis, it was seen that meat neophobia was the unique factor that significantly predicted (not) eating the Bugbar

de Koning et al. [68]	2020	China, USA, France, UK, New Zealand, The Netherlands, Brazil, Spain, Dominican Republic	3091 (M 38.9% F 59.2% no answer 1.9%).<br>Age range: 16-83 years	Quantitative (questionnaire survey)	Food Neophobia; Food Technology Neophobia; Healthiness Influence; Environmental Impact Influence; Meat Nutritional Importance; Meat Taste, Texture, and Smell Importance; Plant-Based Protein Suitability/Benefits; Plant-Based Protein Willingness to Try, Buy, and Pay More; Insect-Based Protein Suitability/ Benefits, Insect-Based Protein Willingness to Try, Buy, and Pay More	Plant-based protein and insect protein meat substitutes

	**Main outcomes**<br>Food Neophobia and Food Technology Neophobia inhibit consumer willingness to try, buy, and pay more for meat-alternative proteins. Meat nutritional importance inhibited willingness to adopt plant-based substitutes and negatively influenced the perceived suitability/ benefits of both meat substitutes. Meat taste/texture/smell importance inhibited willingness to adopt meat substitutes and negatively influenced the perceived suitability/benefits of only insect-based substitutes. Environmental impact of food choices positively influenced perceived the suitability of meat substitutes. Healthiness of food choices positively influenced the perceived plant-based meat substitutes. Meat nutritional importance and plant-based suitability/benefits are main predictors of willingness to try, buy, and pay more for plant-based substitutes. Plant-based responses were significantly higher than the insect-based responses					
Elorinne et al. [95]	2019	Finland	567 (M 33% F 67%)<br>Age range: (not clearly reported)	Quantitative (online survey)	Personal eating habits (omnivore, vegetarian, vegan); Attitudes; subjective norms; Perceived behavioral control; Food Neophobia toward the consumption of insect-based foods intentions towards food choices	Insect-based food (general reference)
	**Main outcomes**<br>Three dietary groups (omnivores, non-vegan vegetarians & vegans). They exhibited significantly different intention to eat foods of insect origin. Vegans held the most rigid negative attitude, and their subjective norm to eat insects was weaker compared to that of omnivores and non-vegan vegetarians. Vegans perceived behavioural control over their eating of insects was stronger compared to that of omnivores and non-vegan vegetarians, and they were more neophobic than omnivores and non-vegan vegetarians. Non-vegan vegetarians held the most positive attitude toward eating insects, and both non-vegan vegetarians and omnivores thought that insect consumption is wise and offers a solution to the world's nutrition problems. In contrast, vegans regarded insect consumption as immoral and irresponsible					
Gómez-Luciano et al. [70]	2019	Spain/ Dominican Republic	401 (201 Dominican Republic and 200 Spain) (M 50.5% F49.9) for Spain.<br>Age range: 16-83 years	Quantitative (questionnaire)	Attitude toward food technologies; Technophobia; preference; Willingness to eat	Plant based proteins, insect-based foods (general reference)
	**Main outcomes**<br>The results show that plant-based proteins are the best positioned alternative, while insects are the worst positioned in the Dominican Republic. Gender and education in the Dominican Republic and gender, education and age in Spain are significant factors for the adoption of alternative to meat proteins. Health and convenience attitudes may determine the adoption of alternative dietary proteins in Spain and the Dominican Republic					
Gumussoy et al. [100]	2021	UK	80 (study1), 78 (study2) (M 23.3% F 57.7%)<br>Age range: 18-24 years	Quantitative (questionnaire and sensory evaluation)	**Study 1**- Independent variables (food content information): Control, Irrelevant inf. (herb mix), Sustainability (chickpea), Delicacy (dried mealworm flour). Dependent variables: tactile sensitivity liking, desire to eat, food intake.	'Cauldron' falafel' and 'Sainsbury's Plain Folded Flatbread'

					**Study 2** - Independent variables: Control, Mealworm and Mealworm + education. Dependent variables: The same of study 1 + latency to eat and self-report disgust.	
	**Main outcomes**<br>**Study 1.** Liking for and desire to eat were lower, and tactile sensitivity somewhat higher, in the Irrelevant-food content information condition compared to the Sustainability condition. By contrast, liking and desire to eat were equally low, and tactile sensitivity equally high, in the Irrelevant-information condition and Delicacy condition.<br>**Study 2.** Information on sustainability and nutritional advantages entomophagy failed to reduce disgust - falafel intake, liking and desire to eat were decreased. However, a passage which described how mealworm flour is produced, did significantly reduce disgust					
Hartmann et al. [71]	2015	China & Germany	502 (from Germany) (M 48% F 52%)<br>Age range: 20-69 years	Quantitative (questionnaire)	Food neophobia; Willingness to consume insects (raw and transformed); Previous consumption of insects	Insects raw & transformed: crickets (deep-fried), silkworm (deep-fried), silkworm drink, cookies based on cricket flour, chocolate chip cookies based on cricket flour
	**Main outcomes**<br>Germans reported higher willingness to eat the processed insect-based foods compared to the unprocessed foods. Further results revealed that low scores for food neophobia, positive taste expectations, high scores for social acceptance and experiences with eating insects in the past were significant predictors of consumers' willingness to eat insects in both countries					
Hartman & Siegrist [87]	2016	Switzerland	104 (M 48.5% F 41.5)<br>Age range: 18-65 years	Quantitative (questionnaire + sensory evaluation)	Willingness to eat; Food Neophobia Scale; Previous consumption; Disgust (for animal-based contamination)	Tortilla chips, one made with a traditional corn flour recipe, another that includes cricket flour as an ingredient
	**Main outcomes**<br>Participants assigned to the experimental condition (tortilla-shaped insect chip) reported a higher willingness to eat unprocessed insects compared to a control group. Greater willingness to eat unprocessed insects among people who had consumed insects in the past and who had low food neophobia scores. People who reported strong feelings of disgust when confronted with living contaminants in food were less likely to be willing to eat insects compared with people who reported a low level of disgust					
Herbert & Beacom [56]	2021	Ireland	105 (M 33% F 67%)<br>Age range survey: 18-24 years<br>Age range focus group: 20-62 years	Qualitative + Quantitative (online focus groups + online survey)	Survey: Snacking habits; Preferences, and opinions related to insect-based food products; Food Neophobia; Likeliness to purchase.<br>Focus group: barriers and drivers of acceptance	Photo of insect-based snack

**Main outcomes**<br>Part of the consumers sample would be willing to try the products, while others would not be open to try them. The insect-based snack consumers were most likely to purchase was a protein bar, crisps and crackers, while the insect-based snack they were least likely to purchase was whole insects. Potential barriers were unfamiliarity, cultural norms, hygiene, or safety concern. Potential factors favouring acceptance were also identified, indicating barriers to consumption of these products could be overcome through product design, education, and effective marketing strategies						
House [104]	2016	The Netherlands	33 consumers of insect-based convenience foods. Gender and age range not reported	Qualitative (individual in person interview)	Willingness to eat insects	Insect-based convenience foods available on the market (burgers, nuggets, schnitzel and 'pittige punten, all prepared with 13-15% ground-up buffalo worms, the larvae of the *Alphitobius diaperinus* beetle)
**Main outcomes**<br>Disjuncture between the initial motivations behind purchasing insect-based convenience foods and the factors affecting repeat consumption. The initial motivations included a general interest or curiosity, a feeling that Insect products are more environmentally friendly or sustainable than conventional meat products, a feeling that Insect products were good for one's health, and/or the introduction of novelty and variety into diets. Factors affecting repeat consumption of insect-based food are price, taste, availability, and 'fit' with established eating practices						
Iseppi et al. [103]	2021	Italy	506 (M 41.5% F 59.5%) Age range: not clearly indicated (the most represented age group is 20–30 years)	Quantitative (online survey)	Openness to try new food; propensity to entomophagy; Personal eating habits (omnivore, vegetarian, vegan, other); Liking / Level of propensity for acceptance	Insects and insect-based foods (general reference)
**Main outcomes**<br>82.8% of the sample declared to have never eaten an insect-based meal; 10.5% consumed insects once or twice at most and the remainder (7%) consumed insects regularly. The research applied the Rasch models and showed that, in case of hunger or need, 70.8% of the sample declared that they would be willing to eat insects. The willingness to habitually consume and pay for insect food is very low, but the percentages are higher than people who had insect tasting experiences. Those who are inclined to consume a beetle showed a maximum propensity for entomophagy, followed by the consumption of locusts. The propensity towards other insects as food, mealworm, and silkworm, was lower. Still lower was the propensity for the consumption of ants, while the lowest propensity score which therefore indirectly indicates a greater consumption, is for crickets and grasshoppers- The lower the value of the propensity score is, the greater is consumption						
Jensen & Lieberoth [75]	2019	Denmark	203 (M 21.7% F 78.3%) Age range: not clearly defined (students)	Quantitative (online survey + tasting evaluation)	Neophobia; Pathogen Disgust Scale; Perceived Infectability scale; Previous consumption; Liking, Willingness to eat insects in foods; Perceived social norms	Spring rolls with visible mealworms, Spring rolls with invisible mealworms, Buttermilk soup with visible mealworms, Buttermilk soup within visible mealworms (edible

						insects: roasted mealworms Tenebrio molitor larvae)

Main outcomes
53.4% participants indicated they would try mealworms if they were offered to them. In the tasting session, however, 81.0% tasted the mealworms. Participants' responses to the food pictures showed that insect visibility had a significant effect. Compared to spring rolls with invisible mealworms, spring rolls with visible mealworms were rated as significantly more inappropriate, significantly more distasteful, and significantly less edible. Ratings of the traditional Danish buttermilk soup followed the same pattern. Self-reported trait-level Pathogen Disgust and Perceived Infectability did not consistently predict insect eating disgust, willingness to eat insects, or actual insect tasting behaviour in the tasting session. In contrast, perceived insect eating norm emerged as a significant predictor of insect tasting behaviour suggesting that perceived social norms play a substantial role in Westerners' (un)willingness to eat insects. The more participants thought other participants ate the mealworms, the more likely they were themselves to eat the mealworms

Jones [106]	2020	UK	187 (Gender not reported)<br>Age range: 7-14 years	Quantitative (questionnaire) + Whole-class workshops with tasting (facial expression/body language) + Qualitative (focus group)	Liking, Willingness to eat food with insects	Burger and Bolognese containing insects of plant protein called VEXo

Main outcomes
80% of young people stated in the questionnaires that they wanted to learn more about sustainability; however, the analysis highlighted three dilemmas that young people face when faced with the consumption of food with insects: uncertainties about the possible health impact of their consumption; questions about the origin of the insects and how they were raised; and concerns about what they would be asked to taste.

Jones & Beynon [105]	2020	UK	124 (Gender not reported)<br>Age range: 6-14 years	Qualitative (focus group) Quantitative (questionnaire + sensory evaluation)	Liking, Willingness to eat food with insects	Burger and Bolognese containing insects of plant protein called VEXo

	**Main outcomes**<br>100% of focus group attendees did not wish to see actual parts of insects in their food but preferred the processing and presentation of plant protein called VEXo in familiar formats (bolognese and burger). Before tasting, only 27% of young people reported they would consider choosing edible insects for a lunchtime option at school. After tasting the product, 74% of young people were positive about its taste, with 54% of those reporting it was tastier than expected. One hundred percent of comments relating to the taste of VEXo bolognese were positive, with all negative comments on taste referring to the burgers					
Kane & Dermiki [88]	2021	Ireland	268 (M 46.6% F 53.4%)<br>Age range: ≥ 21 years	Quantitative (online survey)<br>Qualitative data from open questions	Attitudes towards the health and nutritional benefits of meat, towards the health characteristics of food; Attention to environmental impact of food choices; Food neophobia; Openness to try new foods; Willingness to try insects.	Food with insects (general reference)
	**Main outcomes**<br>More men (57.4%) were willing to try insects compared to women. No effect of education level, course type and age on willingness to try. No effect of age was observed in the willingness to try. There was a significant effect of diet, as higher percentages of vegetarians (80%) and vegans (100%) were not willing to try insects, whereas among those who do not following a specific diet slightly more than just over half were willing. In terms of the diet-related behaviour, there was no effect on the meat score, environment score or health scores; however, there was a significant effect on the food neophobia score (people who were not willing to try insects had higher scores) for food neophobia compared to those who were willing to try insects. Previous experience significantly affected willingness to try insects: (67.5%) would try them again, whereas among those who had not tried insects before only 49.6% were willing to try them. The first condition for trying insects was that were "tasty", followed by nutritious and safe. Insects disguised in familiar foods are the most likely to be accepted than whole insects					
Kornher et al. [89]	2019	Germany	311 (M 26.8% F 73.2%)<br>Age range: not reported (adults)	Quantitative (online based choice experiment + quantitative questions)	Choice experiment (Attributes: Production method - conventional vs. organic-, health claim, visual impression, carbon emissions, composition of the burger patty, and the purchase price)<br>Quantitative variable: Aversion towards eating insects; Disgust; Readiness to eat insects; Previous consumption; Preferences for learning about food products and recipes; Food neophobia; Aversion towards eating unfamiliar food products; Attitude towards organic production method; CO2 Emissions Attention; Preference for convenience food; Importance of nutritional information; Importance of taste	Burger fortified with pulverized insects
	**Main outcomes**<br>The largest group of respondents is willing to consume insect-fortified burgers with only a small price discount, while the other respondents had a prohibitively low willingness-to-pay. The readiness of consumers to adopt insects into their diet is strongly related to attitudinal variables, such as preferences					

	for an environmentally friendly production method and health aspects. On the other hand, disgust, food neophobia and the aversion towards insects seem to be the main reasons to abstain from eating insects					
Kostecka et al. [97]	2017	Poland	210 (M 50% F 50%)<br>Age range: ≥18 years	Qualitative (online questionnaire with closed answers)	Consumers' opinions related to their acceptance of insect-based food as an alternative source of nutrients	Insect-based food (general reference)
	**Main outcomes**<br>Representatives of Polish consumers in the region of Podkarpackie generally did not show open-mindedness towards incorporating insect-based food into their diet or even use of insects as animal feed. The thought of eating insects was disgusting for over 40% and nauseating for 39% of the survey participants. Majority of the respondents, however, recognized the importance of food sector operation based on respect for natural resources					
Kouřimská et al. [74]	2020	Czech Republic	98 (M 18,4% F 81.6%)<br>Age range: 18-45 years	Sensory evaluation	Liking	House cricket
	**Main outcomes**<br>Sensory analysis of home-cooked crickets showed a significant difference in the hedonic evaluation of overall pleasantness before and after tasting of the samples with more negative score before tasting. About one third of the participants were not willing to try house crickets. Women and younger assessors were slightly more open to entomophagy. No statistically significant differences were found between males and females or between the two age categories in the assessment. The preferred culinary preparation of edible insects that the respondents would choose was baking, roasting, and frying					
La Barbera et al. [83]	2018	Italy/Denmark	118 (M 50.8% F49.2%)<br>Age range: (not clearly defined - students)	Qualitative & quantitative approach (experiment+ self-assessment + interview)	Neophobia; Disgust; Intention to try new food liking; Preference; Indirect measures (Implicit Association Test)	Insects as food ingredient (Chocolate bar with crickets' protein)
	**Main outcomes**<br>Both food neophobia and disgust make independent contributions to the intention to eat insects, and the explanatory power of disgust is considerably higher. Moreover, a significant effect of implicit attitude on disgust and an indirect effect of implicit attitude on intention mediated by disgust have been found					
La Barbera et al. [86]	2019	Italy	280 (M 43.2% F 56.8%)<br>Age range: ≥ 18 years	Quantitative (online questionnaire)	Disgust; Food Neophobia; Intention to eat and to introduce in the ordinary diet raw insects, processed insects and other animals fed with insects	Foods based on row insects, processed insects, or insects as feed

	**Main outcomes**<br>The influence of FNS on the intention scores was different --> food neophobia tends to increase when moving from direct to indirect entomophagy and to decrease when moving from regular to occasional consumption. There are no differences between intention to try or consume raw insects on a regular basis --> the intention scores are very low. More favourable intention for processed insects and indirect entomophagy. Disgust is a better predictor of intention to introduce raw insects in the diet					
Lammers et al. [90]	2019	Germany	516 (M 48.4% F 51.6%)<br>Age range: 18-87 years	Quantitative (online questionnaire)	Eating habits (diet, meat consumption, intention to reduce meat intake); sensation seeking; sustainability consciousness; food neophobia; food technology neophobia; food disgust; previous insect consumption; willingness to try insect-based food; sociodemographic characteristics	Insect burger and buffalo worms
	**Main outcomes**<br>A total of 41.9% of the participants were willing to consume an insect burger. In contrast, only 15.9% of the participants were willing to consume the buffalo worms – a principal ingredient of the insect burger. Food disgust was the most important predictor for the acceptance of edible insects, followed by previous insect consumption, food neophobia, gender, sensation seeking and food technology neophobia. The high influence of food disgust shows that not only the explicit disgust for insects is decisive for the consumer readiness of insect-based products, but also the domain specific disgust for food. Sustainability consciousness did not prove to be a significant predictor for the willingness to consume insects					
Laureati et al. [76]	2016	Italy	341 (34.6% F 65.4)<br>Age range: 18-80 years	Quantitative (questionnaire + visual hedonic evaluation).	Willingness to incorporate insects into diets; Food Neophobia; Consumers' sustainable behaviour; Expected Liking (visual hedonic assessment)	Picture of foods containing insects or insects derived proteins: Biscuits made with insects, Cereal bar containing insects' flour, Chocolate-coated grasshopper, Cereal bar containing insects, Apple salad containing insects, Tequila containing a larva, Risotto containing maggots, Maggot cheese, lollipops containing larvae
	**Main outcomes**<br>The principal factors affecting the Italian consumers' readiness to adopt insects as food and feed were age, gender, cultural background, and food neophobia. Subjects' involvement in sustainability issues did not play a role in the acceptance of insects. In the hedonic evaluation, biscuits made using insect flour and chocolate-coated grasshoppers were significantly more liked than were the other products. A cereal bar containing insects, an apple salad containing insects and tequila containing a larva were very much disliked; however, they received significantly higher ratings compared with risotto containing larvae, cheese with larvae and lollipops containing larvae					
Mancini et al. [91]	2019	Italy	165 of which 66 taking part to the tasting session (M 39.29% F 39.86%)<br>Age range: not reported (university students)	Quantitative (questionnaires + hedonic scales)	Food neophobia; Beliefs towards the consumption of insects (i.e., disgust, distaste, fear, social acceptance); Previous consumption; beliefs about effect on health, Effect on the environment, Familiarity of	Bread with insects

					taste; Intention to eat insect-based food; behavioural control.<br>Liking for appearance, odour, flavour, texture, overall liking (after tasting); Willingness to eat insects.	
	**Main outcomes**<br>Perceived behavioural control was the main predictor of the intention, followed by neophobia and insect based-food rejection. The disgust factor significantly decreased after the participants attended an informative seminar. Sensory scores showed that participants gave the "insect-labelled" samples higher scores for flavour, texture, and overall liking, However, participants indicated that they were less likely to use the “insect-labelled” bread in the future					
Moruzzo et al. [96]	2021	Italy	420 (M 47%; F 53%)<br>Age range: 19-35 years	Quantitative (questionnaire)	Neophobia; Insect Phobia	Insect-based food (general reference)
	**Main outcomes**<br>The probability of eating food containing insects decreases from 69% (for those who have a low level of neophobia) to 67% (for those who have a low level of insect phobia). The intention to eat food containing insects increases from 51% (for those who have a low level of neophobia) to 54% (for those who have a low level of insect phobia). The probability of eating food containing insects decreases from 24% (for those who have a high level of neophobia) to 21% (for those who have a high level of insect phobia). Similarly, the intention to eat food containing insects decreases from 8% (for those have a high level of neophobia) to 4% (for those who have a high level of insect phobia)					
Modlinska et al. [81]	2020	Poland	99 (M18.2% F 81.8%)<br>Age range: 18-45 years	Quantitative and Qualitative approaches (questionnaires + behaviour assessment + individual interview).	Food Neophobia; General Neophobia Scale; Food Technology Neophobia; Disgust Sensitivity; Variety Seeking Tendency in food choices; Willingness to try; Visual attractiveness of the product; smell; taste; Willingness to eat more of the product; Amount of food eaten.	Insect-based foods with informative labels: cookie, muffin, date ball
	**Main outcomes**<br>The products labelled as "containing insects" are consumed with reluctance and in lower quantities despite their appearance (smooth or with traces of insect-like parts). The effect of Label was found in the latency to pick up food, latency to begin eating, amount of food eaten, and time spent eating variations. Food neophobia correlated only with the amount of food eaten for the date ball. General neophobia and variety seeking tendency correlated with the latency to pick up food. People with lower general neophobia and higher variety seeking tried the insect-labelled samples sooner than people with high food neophobia level. No differences were found regarding the scores awarded for appearance, smell, taste, and willingness to eat more for products labelled/not labelled as "containing insects". In the interview emerged that the first reaction to ingesting insects was disgust. According to the participants, the high nutritional value of					

insects was seen as an advantage, and insects could become a fashionable "curiosity" in some social circles. They imagined that a potential insect consumer was a young person open to new experiences, with a positive attitude, who wants to reduce the amount of meat or to stop eating it

Modlinska et al. [98]	2021	Poland	1096 (M 45% F 55%)<br>Age range: 16-78 years.	Quantitative: (online survey. Computer-Assisted Web Interview)	Attitudes towards health characteristics of food; Familiarity with insect consumption; Environmental concerns; Convenience orientation in relation to food; Willingness to buy; Food Neophobia & General Neophobia, Variety Seeking; Disgust sensitivity; Travel frequency (food experiences abroad)	Insects (general reference) as meat substitute
	**Main outcomes**<br>The analysis showed a significant effect of gender but no effect of age or educational level. Males were 1.8 times more likely to admit that insects can be a substitute for meat. Diet had no effect. Vegetarians, vegans, and omnivores accepted insects as a meat substitute at the same level. None of the psychometric properties (food neophobia, general neophobia, sensitivity to disgust, variety-seeking tendency) had any effect on accepting the idea of insects as meat substitutes. Participants who were familiar with the concept of entomophagy were 1.3 times more likely to accept insects as a meat substitute compared to those who were not familiar or who were less familiar with the idea. Similarly, attention to the environmental impact of food choices was responsible for a 1.3-times higher likelihood of accepting insects as meat alternatives					
Niva & Vainio [99]	2021	Finland	1000 (M 49.5% F 50.5%)<br>Age range: 17-79 years	Quantitative (online questionnaire)	Food Neophobia; General health interest; Natural product interest; Sustainability concerns; Economic concerns; Hedonics (importance that food pleasure has); Changes in intake; Expectations about changes in the future coming years; Eating motives; consumption frequency of protein sources	Insect-based and plant-based products (general reference)
	**Main outcomes**<br>Four behavioural clusters were identified. The largest cluster, *“Established beef lovers”* (37% of the respondents), reported stable consumption of beef, and no consumption of plant- or insect-based protein products and do not intend to make any changes in the consumption of these foods in the future. The cluster "*Alternative protein increasers*" (25.5%), had reduced beef consumption, and intended to decrease it more in the future. The cluster "*Established ‘light’ flexitarians*" (20.3%), reported no changes in the past 2–3 years in the consumption of beef or plant-based protein products, and no consumption of insect-based foods. Nor did the majority in this group intended to make any changes to their consumption in the future. The cluster "*Beef-avoiding plant protein increasers*" (9.3%), reported that they did not consume beef and have no plan to consume it in the future. In contrast, most of them reported having increased the consumption of plant-based protein products, and about half of them intended to increase it in the future. Very few had consumed insect-based foods, and the majority did not intend to consume them in the future					

Nyberg et al. [109]	2020	Sweden	82 (M 35.4% F 64.6%) Age range: ≥ 18 (not clearly specified)	Qualitative (questionnaire + Focus group during a workshop for 15 experts in insects)	Reasons to eat proteins from insects	Insects (general reference)
		**Main outcomes** Environmental sustainability followed by health and exciting were the three most frequent responses regarding reasons for choosing insects as a protein source. More men than women considered crispy as an important aspect. No differences between ages were observed. “Tasty” and “trendy” were both considered minor factors for choosing insects as food				
Orsi et al. [92]	2019	Germany	393 (M49% F 51%) Age range: 13-82 years	Quantitative (online questionnaire)	Food neophobia; Environmental awareness; Health consciousness; Previous experience; Disgust; Acceptance of insects as food; Risk assessment of whole and processed insects; Acceptance of insect food products available in Germany; Willingness to try; Familiarity and perception	One snack containing whole insects, and four processed insect products, namely, aa protein bar; pasta, granola, and an insect burger.
		**Main outcomes** Most of the sample (74%) had never tried insects in any form and 56.7% of the sample were unaware of the availability that insect-based food products in Germany. Food neophobia and disgust had the most significant negative impact on the acceptance of whole and processed insect products. Health-conscious consumers are keener to accept processed insect-based products. Furthermore, this target group was open to new, functional food alternatives. In relation to sociodemographic factors, women had higher aversions to eating whole insects than men, but not in relation to processed insect foods. Young non-vegetarian males were more likely to accept insects as food. People with higher environmental awareness were more likely to try insects as an alternative food source				
Palmieri et al. [101]	2019	Italy	456 (M 32% F 68%) Age range: 18-65 years	Quantitative (online questionnaire)	Diet type (vegetarian, vegan, omnivore); Meat consumption; Attitudes towards healthy food; Attitude towards collecting information; Attitude towards sustainable food, Neophobia; Openness towards new foods; Food Technology Neophobia; Willingness to try; Knowledge about insects, Previous experience; Perceived risk	Insect-based food (general reference)

	**Main outcomes**<br>83% of the respondents are not inclined to eat insects. For 13% insects are associated with a sense of danger. Consumers who care about food sustainability and collective health are, on average, more likely to be willing to consume insects. Those who have a positive attitude towards new food are more likely to eat insects. Those who reported previous experiences with insect-based food have 10 per cent more probability to eat it again in the future; those who indicated precise aspect and taste expectations as influential features in their decision are 22–23% more likely to adopt insect in their diets. Those who do not believe that eating insects can be dangerous for their health show on average a propensity to try insect-based food, which is about 3% higher than those with opposite beliefs. Women are on average about 4% less likely to be inclined to eat insect-based food than men. Age exerts a negative effect on the probability to be willing to eat insects: the estimated probability that a 50-year-old individual is attracted by insect-based food is on average about 1 per cent less than for a 30-year-old one					
Russo et al. [80]	2020	Italy	Study 1:<br>1285 (M49.6% F 50.4%)<br>Study 2:<br>44 (M 45.5% F54.5%)<br>Age range: 18-29 years	Study 1<br>Quantitative (questionnaire)<br>Study 2<br>Neuro-psychophysiological data	Study 1 - Adaptation of Food Choice Questionnaire.<br>Study 2 - Neuro-psychophysiological data, namely EEG, skin conductance and eyes movements	Crackers made with grain flour and the same crackers labelled, "Made with earthworm flour" with two claims: an healthfulness claim or a tastiness claim
	**Main outcomes**<br>**Study 1**. Determinants of food choice: "sensory" and "health" claims are key drivers during food purchasing crackers labelled "Made with earthworm flour". Familiarity seems not to play a relevant role, denoting young consumers' openness to novel food. A significant difference was found between the two communication strategies, as the claim about healthfulness was most effective provoked a greater approach to the product, especially among males. In general, females showed a greater rejection to this novel food.<br>**Study 2**. Effect of Labels. The effect on the skin conductance level was significant. There was a significant difference between "grain flour" and "earthworm flour" labels both for tastiness and healthfulness claims. The neurophysiological response of the subjects while watching the images of earthworm flour crackers, did not show any effect, neither of gender nor of claim, while a significant interaction effect between the two variables was found. In particular, the claim focused on healthfulness led to a stronger response in comparison with the claim on taste. Females tended to keep a higher distance from the screen than did males while watching the image of the crackers made with earthworm flour, independent of the claim. The reaction measured by EEG also differed for gender when claims were for healthiness					
Schäufele et al. [93]	2019	Germany	342 (Gender distribution not reported)<br>Age range: not reported	Quantitative (questionnaire)	Food neophobia; Familiarity for insects as foods; Social acceptability for insects; Preference, Willingness to try; Predicted taste and flavour; Insects' perceived benefits,	Pictures of dishes (Rice with chives and tomatoes, with 2 species of insects and 3 levels of visibility: grasshoppers and mealworm/ whole insect, crushed insects and insects processed as meatballs)

		**Main outcomes** The appetising appeal and the willingness-to-try the carrier dish was the best graded option compared to all the other dishes. As for dishes containing insects, participants were, in most cases, "not willing at all" to try them. Social acceptability was the predictor with the highest influence on the willingness-to-try. This means that with a higher perception of entomophagy as a civilised activity, the willingness-to-try the insects increased. In a second instance, "food neophobia" also showed a huge effect. As for socio-demographic variables, only "gender" showed a significant effect; female participants rated willingness-to-try more negatively. The results suggest that dishes containing Mealworms or whole insects are less preferred than the ones containing grasshoppers or processed insects. The variable of species had a medium effect on the variance of the willingness-to-try when they were whole insects, and a small effect when they were crushed or processed as meat. Thus, for whole insects, the negative effect of Mealworms on consumer acceptance was much greater compared to other degrees of visibility				
Sogari et al. [82]	2019	Italy	88 (M 48.9% 51.1%) Age range: 18-40 years	Quantitative (questionnaires and sensory analysis)	Food neophobia; Previous insect consumption; Sensory property expectation; Intention to eat an insect product; Sensory evaluation of insect products	Insect (as general reference in the questionnaire) and visible insect product (whole cricket in a jelly sweet) and the insect-based product (a cricket flour in a jelly sweet (for tasting)
		**Main outcomes** The results showed moderately positive sensory expectations of eating insect products, both in terms of appearance and taste. The respondents reported moderately positive intentions to try unprocessed insect products, while the intention to try processed insect-based products was significantly higher. People who show openness to try new foods (i.e., ethnic products from different countries) will be considered as less neophobic and will tend to try unfamiliar products (i.e., edible insects) more easily. Past exposure to insects is positively related with sensory expectations about insects and negative related with neophobia, at the same time, the food neophobia contributes negatively to the intention of eating insects, whereas the sensory expectations contribute positively. In this model, it is observed that gender has an effect and male are more prone to eating insects than female. Age negatively affects the intention to eat insects, with older people being less open to that				
Tan et al. [72]	2015	The Netherlands and a country outside Europe (Thailand)	54 (M 35.2% F 64.8) Age range: 20-65 years	Qualitative (semi-structured focus-group); Quantitative (liking scale)	General dietary preferences; Openness to trying new foods; Individual experiences and knowledge; Reasons to eat or not eat insects; Evaluation of images of insect species and products (liking); Optional tasting and evaluation of insect-based products	Eight whole insects (ant larvae, big-butt ants, grasshopper, giant water bugs, mopane worms, witchetty grubs, mealworms, bamboo worms) and 6 insect-based foods: 2 visible (fried grasshopper with chilly and salt, mealworm muffin with chocolate pieces, 2 covered (cricket fritters with roasted peanuts, chocolate-coated grasshoppers,) 2 invisible (giant water bug chili paste, butter cookies with ground beetles)

Main outcomes
Dutch participants not having the same cultural exposure to insects as food as the Thais, demonstrated a high level of interest, mainly motivated by the novelty of the experience, the environmental and health benefits of eating insect and their interest in the search of sustainable and nutritious alternatives to meat, while the Thai participants considered insects more in terms of their taste and familiarity. Cultural exposure created expectations of which species were more appropriate to eat and how they should be prepared. Presenting insects in a recipe context generally resulted in more positive evaluation. However, reducing the visibility of insects did not necessarily improve liking of a product, where fried grasshoppers were preferred over chocolate-coated grasshoppers. After receiving insect-based products to taste, the final decision to taste was a mixture of interest to try and fear or disgust. After tasting, many Dutch participants remarked that they were not yet ready to accept insects as regular food even if sensory expectations were satisfied as other practical food concerns were still a barrier to acceptance

Tan et al. [107]	2016	The Netherlands	103 (M 60.2% F 39.8)<br>Age range: not reported	Quantitative; (participants first see images of a grilled beef burger and are requested to imagine that they are composed of different novel foods; labels were presented)	Food neophobia; Sensory liking before and after tasting; Food appropriateness; Willingness to eat; Familiarity for the novel foods considered	Four beef-based burgers (with plant-based ingredients to create sensory differences between formulations) combined with four label descriptions (100% beef or 75% beef and 25% lamb brain, 25% frog meat or 25% mealworms)

Main outcomes
Before the tasting, label significantly influenced both expected sensory-liking and food appropriateness, where novel burgers were rated significantly less positively than the beef burger. After the tasting, label did not have a significant main effect on the level of sensory-liking, where novel burgers were evaluated to be similar in sensory-liking as the beef burger, whereas the level of food appropriateness of the novel burgers remained lower than that of the beef burger. Willingness to eat remained lower than that of the beef burger. Males and more neophilic participants evaluated the burgers more positively on sensory-liking and food appropriateness than females and neophobic participants. Although food neophobia and gender effects were significant, their effects were small in comparison to the label effects. Effects of gender were slightly stronger before tasting than after tasting, where the taste experience reduced the differences in evaluations between male and female participants. Willingness to eat was predicted mainly by food appropriateness, and not by the experienced sensory liking nor the individual traits (food neophobia, gender)

Tan et al. [108]	2017	The Netherlands	100 (M 66 %; F 34%)<br>Age range: not reported	Quantitative (questionnaire)	Food neophobia; Image presentation and expected sensory properties using Rate-All-That-Applies (RATA); Food appropriateness; Sensory evaluation; Future willingness to eat (3 situations: meal, special occasion, and snack)	Mealworms beef burger; Lamb brain burger; Frog meat burger; Mealworm burger'

Main outcomes
Presenting the novel ingredients within a burger patty reduced the expected intensities of negative attributes (e.g. bitter, slimy). The expected sensory profiles of the novel ingredients differed significantly between participants who have and have not tasted it before. Participants who have never tasted the novel ingredients before seemed to associate more negative attributes than those who have tasted them before. Labels had a small but significant effect on some attributes: samples were evaluated as mealier when labelled as a mealworm burger. The recipe used also had effect

Tan et al. [84]	2016	The Netherland	976 (M 33.6% F 66.4%)<br>Age range: 18-94	Quantitative (questionnaire presentation of images; scales)	Food neophobia; Preferences regarding the preparation of mealworms; Expected sensory liking; Familiarity; Perceived appropriateness of mealworms as food	Pictures of Mealworm invisible (Beef stew with ground mealworms, Curry with ground mealworms, Brownies with ground mealworms, Spice cake with ground mealworms) Pictures of Mealworm visible (Beef stew with whole mealworms, Curry with whole mealworms, Brownies with whole mealworms, Spice cake with whole mealworms)
	**Main outcomes**<br>Prior taste experience was related to higher ratings of liking of carrier products. Results suggest that reducing insect visibility might be sufficient to lower the barriers of trying products. Food neophobia scores significantly influenced acceptability, where higher food neophobia score was significantly related to lower acceptability ratings. Gender effects were significant across all acceptability measures, where male participants were consistently more positive than female participants in their evaluations. Gender differences were mainly at the level of willingness to try. Younger participants were more willing to try, older participants were slightly more positive in liking and buying, but age effects were not significant. Overall, the effects of the socio-demographic variables on the acceptability measures were absent or small					
Vartiainen et al. [102]	2020	Finland	567 (M 33.2% F 66.8%)<br>Age range: (not clearly reported)	Quantitative (online questionnaire)	Neophobia; Health concern; Willingness to buy/eat	Insect-based food (general reference)
	**Main outcomes**<br>The authors found that women, students, those under 25 years of age, those living in rural areas and those who had no earlier experience of eating insects had less intention to consume insect-based foods. Based on the respondents' perceptions of conditions for the consumption of insect-based foods, three clusters of consumers were identified: 'likely consumers', 'potential consumers' and 'unlikely consumers'					
Verbeke [94]	2015	Belgium	386 (M 39% F 61%)<br>Age range: 18-79 years	Quantitative (questionnaires - scales)	Food neophobia; Food technology neophobia; Attitudes towards health; Convenience orientation; Attention to environmental impact; Belief in the health and nutritional benefits of meat; Consumers' focus on taste or the sensory experience; Consumers' readiness to accept insects; Awareness about the eating of insects	Insects as meat substitute (general reference)

	**Main outcomes**<br>The most likely early adopters of insects as a novel and sustainable protein source in Western societies are younger males with a weak attachment to meat, who are more open to trying novel foods and interested in the environmental impact of their food choice. Age was associated with a decrease in the likelihood of being ready to adopt insects as a food, while familiarity with the idea of eating insects was associated to an increase compared to who had never heard about the eating of insects. Food neophobia and food technology neophobia were both significant determinants of consumers' readiness to adopt insects as a meat substitute. The orientation towards convenience and the importance attributed to the environmental impact in food choices strongly increased the likelihood of being ready to adopt insects. The effect of a person's health orientation was only marginally significant. Adoption of insects to replace meat results 4,5 time higher for who intend to reduce their intake. The importance of the taste of meat sensibly decreases the predicted probability of being ready to adopt insects as a meat substitute					
Verneau et al. [69]	2016	Denmark and Italy	282 (M=50.7% F=49.3%)<br>DK 141 (M=53.9 F=46.1)<br>IT 141 (M=47.5% F=52.5%)<br>Age range: not clearly reported (students)	Qualitative (Implicit Association Test)<br>Quantitative (scales)	Implicit association test (IAT test) (categorize stimuli belonging to the target categories (Insect or Flower) and stimuli belonging to two opposite attribute categories (Positive and Negative); Familiarity with insects as foods; Openness to try insect; Acceptance (2 weeks after having the opportunity of tasting)	Insects (general reference)
	**Main outcomes**<br>Previous knowledge (familiarity) was significantly correlated with intention but not with behaviour. The main effect of the message passed through a video, on intention on the day of the experiment, was significant, with the mean intention score higher for the *social benefit group* (who saw a video on social benefits of introducing insects' proteins into human diet) and the *individual benefit* group compared to the control group (who saw a neutral video on benefits of introducing tablets in schools). While the two types of messages – about individual and about social benefits – had similar effects when intention was measured immediately after exposure, the effect of the information on social benefits appeared to be more stable over time. Effect of familiarity was observed at the time of exposure and 2 weeks after, with familiarity being positively associated with the effect of the message. Effect of country on intention was also significant, the mean intention score was higher for the Danish than Italians. Gender also had effect, but only in the day of the experiment, with message leading to more acceptance in males					
