# Supplementary material for: Determinants of Consumers’ Acceptance and Adoption of Novel Food in View of More Resilient and Sustainable Food Systems in the EU: A Systematic Literature Review
Source: Foods. 2024 May 15;13(10):1534. doi: 10.3390/foods13101534 (PMC11120339; doi:10.3390/foods13101534)
Supplement: Supplementary file 1 [file foods-13-01534-s001.zip › Supplementary Table S3.pdf]

Table S3. Characteristics of the studies on other products of other animal origin (different from insects).

Authors	Year	Country	Participants' number and characteristics	Type of approach	Assessed variables	Type of product
Beriain et al. [116]	2020	Spain	180 (M 39%; F 61%)<br>Age range: ≥ 20 years	In-home use test (questionnaires)	Meat purchase frequency; Sensory analysis (visual, textural and flavour aspects; hedonics); Willingness to pay	Meat enriched with nutritional ingredients (vegetable oil mixture and vitamin D3)
	**Main outcomes**<br>Sensory aspects (taste and freshness) are the main determinants; Age, educational and economic level (people over 65 with a higher level of education and economic level who best valued the characteristics of the modified patties) are also determinants; information about the product is important, particularly for women					
Boereboom et al. [113]	2022	Multi-country (France, Netherlands, Spain, UK)	1291 (M 28.7%; F 71.3%)<br>Age range: ≥ 18 years	Quantitative (online questionnaire)	Intention of eating meat in the future; Food neophobia; Food technology neophobia; Importance of healthiness on food choices; Love for cooking; Attention for the environmental when making food choices	Cultured meat
	**Main outcomes**<br>Differences between countries with Spain and France having lower willingness to engage with cultured meat; Food neophobia and food technology neophobia are barriers for the acceptance of cultured meat					
Broeckhoven et al. [115]	2021	Multi-country (Poland, Finland, Netherlands, UK, Spain)	2500 (M 52.3%; F 47.7%)<br>Age range: ≥ 65 years	Quantitative (online questionnaires)	Dietary habits, intention to change protein sources, food-related attitudes; knowledge/perception of protein in the diet; Food related attitudes; knowledge/perception of protein and their relative environmental impact	Protein-enriched burgers
	**Main outcomes**<br>Gender, country, health, sustainability and familiarity, knowledge of protein and the environmental impact of different protein sources are determinants for the acceptance of protein alternatives by the elderly; engagement to meat (meat lover) is a barrier to acceptance. Communicating healthiness of these products can increase acceptance in the elderly; Carbon labels were proposed a way of improving acceptance in elder adults.					

Dupont et al. [114]	2022	Germany	497 (M 50.1%; F 49.9%)<br>Age range: 18-86 years	Quantitative (online questionnaire)	Eating habits (diet, meat consumption, intention to reduce meat consumption), nutritional-psychological variables (food disgust, food neophobia, food technology neophobia, sensation seeking, and green consumption values), knowledge of cultured meat as a foodstuff (familiarity, previous consumption), (intention to consume a cultured meat burger, attitudes towards cultured meat, specific attitudes towards a cultured meat burger, perceived behavioural control, and subjective norm)	Cultured meat
**Main outcomes**<br>The perceived unnaturalness associated with cultured meat is a barrier; food technology neophobia is a barrier; food disgust proved to be a negative predictor for general attitudes towards cultured meat; Perceived behavioural control was the strongest predictor of willingness to consume a cultured meat burger; association to environmentally sustainable is a driver; age and gender had no significant influence; no influence of meat consumption on willingness to consume a cultured meat burger could be demonstrated. Communicating with words "clean meat" or "animal free meat", or others reducing the association with "artificial" are suggested as strategies to increase acceptance						
Dupont and Fiebelkorn [111]	2020	Germany	718 (M 42.5%; F 57.5%)<br>Age range: 9-19 years	Quantitative (in-person questionnaire)	Dietary habits (diet, meat reduction); Nutritional-psychological factors (food disgust, food neophobia); consumption of novel foods from animal origin (insect or cultured meat, either as food or burger)	Cultured meat
**Main outcomes**<br>Cultured meat was rated more positively when considering the factors ethics, animal welfare, and disgust. Perception of artificiality was a barrier; perception of cultured meat as disgusting is a barrier; The attitude toward the food was the second most important influencing factor for the willingness to consume the cultured meat burger; Age is a determinant, with older adolescents more willing to consume; Food neophobia was also a barrier; No effect of gender or familiarity						

Piochi et al. [112]	2022	Italy	603 (M 39%; F 61%) Age range: 15-80 years	Quantitative (online questionnaire)	Habits related to meat consumption (frequency of consumption of meat, reasons for not consuming traditional meat, degree of knowledge of cultured meat); personality traits (food neophobia, disgust sensitivity, and attitude to cultured meat (favour, willingness to try, willingness to purchase, willingness to substitute for traditional meat)	Cultured meat
	**Main outcomes** Curiosity is a driver; Food neophobia is a barrier; In the present study, disgust sensitivity was not related with cultured meat acceptance; Positive information and claims about environmental impact are drivers. Males are more willing to try, as well as individuals with less than 30 years old					
Profeta et al. [117]	2021	Germany	500 (M 49%; F 51%) Age range: ≥ 18 years	Quantitative (online questionnaire)	Preferred buying location of meat products; Buying frequency of organic free range meat; Food neophobia; Habitual consumption of meat alternatives; Perception of meat hybrids	Meat hybrids
	**Main outcomes** Familiarity (unfamiliar) is a barrier to accept meat hybrids; Neophobia is a barrier; Meat attachment also represents a barrier for diet change and transition					
Siegrist and Hartmann [110]	2020	Multi-country (non-Europe – Australia, US, Mexico, China, South Africa; Europe - UK, Spain, France, Germany, Sweden)	6128 (M 49% - 52%; F 48% to 51%) Age range: 20-69 years	Quantitative (online questionnaire)	Food disgust sensitivity; Food neophobia; willingness to eat cultured meat; Trust in stakeholders (food industry)	Cultured meat
	**Main outcomes** Cultural background is a determinant (a country like France shows lower acceptance food cultured meat); perceived naturalness has a direct positive effect on the acceptance (although the effect is different for different countries); Food neophobia and food disgust sensitivity are barriers to acceptance (except France).					

Tan et al. [107]	2016	Netherlands	103 (M 60.2%; F 39.8%)<br>Age range: 22.9 ± 5.5 years	Quantitative (in-person questionnaires)	Sensory analysis (hedonic liking; descriptive sensory evaluation); Food appropriateness; Familiarity; Food neophobia	Lamb brain and frog meat
	**Main outcomes**<br>Food appropriateness is determinant (the quality of taste experience is important to learn about food appropriateness); Food neophobia is a barrier; males are more neophilic; curiosity (situations for experiences) is driver for higher acceptance.					
Tan et al. [108]	2017	Netherlands	100 (M 66%; F 33%)<br>Age range: 23.3 ± 6.3 years	Quantitative (in-person questionnaires)	Expected sensory properties using Rate-All-That-Applies (RATA) checklists (after see images); Sensory evaluation (at tasting); willingness to eat in future; Food appropriateness; Familiarity; Reasons for not tasting	Lamb brain and frog meat
	**Main outcomes**<br>Previous experience is a driver (taste experience is able to correct misconceptions of these food products); Appropriateness is more important than taste to willingness to eat; To present these products without visualizing their original aspect is positive; the expectation of a taste equal to the original product (meat) is negative, since results in desolation					
