# Supplementary material for: Determinants of Consumers’ Acceptance and Adoption of Novel Food in View of More Resilient and Sustainable Food Systems in the EU: A Systematic Literature Review
Source: Foods. 2024 May 15;13(10):1534. doi: 10.3390/foods13101534 (PMC11120339; doi:10.3390/foods13101534)
Supplement: Supplementary file 1 [file foods-13-01534-s001.zip › Supplementary Table S4.pdf]

**Table S4.** Characteristics of the studies on plant-based products (M=Males; F=Females).

| Authors | Year | Country | Participants' number and characteristics | Type of approach | Assessed variables | Type of product |
|---|---|---|---|---|---|---|
| Ali et al. [118] | 2021 | Italy, Germany, Netherlands, Finland, other EU countries | 291 (M 32%; F 68%). Age range: 18-75 years | Quantitative (online questionnaire) | Willingness to pay, attitude | Wheat bread, consumer potatoes and tomato sauce obtained with microbial applications to replace synthetic chemical inputs |
| | **Main outcomes** Consumers' WTP is positively affected by the reductions of chemical use, income and health concerns have no statistically significant effects on WTP. Positive associations have been observed between environmental concern and WTP for organic food products; age, gender, education and income are associated with WTP for foods produced with 20% less chemical use. Promotion-oriented Food choice motives construct positively affected WTP for food products that are produced with microbial applications | | | | | |
| Appiani et al. [138] | 2021 | Italy | 120 (M 44.0%; F 56.0%) Age range: 20-60 years | Quantitative (scales) | Liking scores | Corn-based gluten-free formulations added with Tartary buckwheat |
| | **Main outcomes** Liking was positively correlated with a low intensity of dryness, overall flavour, and bitterness, whereas a high intensity of overall flavour, bitterness, yellow/ochre colour, firmness, and dryness were associated with samples disliking. PROP responsiveness and food neophobia were not significantly different between clusters (cluster of black wheat likers and cluster of no likers) | | | | | |
| Broeckhoven et al. [115] | 2021 | Poland, Finland, The Netherlands, UK, Spain | 500; (M 52.3%; F 47.7%) Age range: Older than 65 years | Quantitative (online questionnaire) | Willingness to pay, knowledge and perception of protein-sources | Carbon labelled protein-enriched burgers |
| | **Main outcomes** Protein-enriched burgers are a potentially acceptable product to increase older adults' protein intake but WTP for protein-enriched plant-based burgers is negative. The carbon labels, highlighting the lowest amount of environmental impact in terms of carbon emissions, were only appreciated if referred to red meat or poultry-based burgers but not to plant-based burgers. Plant-based meat is hardly acceptable by red meat consumers meanwhile poultry meat consumers are most likely to shift their protein consumption based on health-related motives. Income, price, convenience, and sensory appeal are not associated with older adults' preferences | | | | | |
| Castellari et al. [137] | 2019 | Italy | 115 (M 40.0%; F 60.0%) Age range: 30-40 years | Quantitative (questionnaires using scales and tasting session) | Willingness to pay | Fruit jam enriched with Aloe vera gel |

| | | | | | | |
|---|---|---|---|---|---|---|
| | **Main outcomes**<br>WTP for the aloe compote was always greater than for the conventional fruit-only compote. Moreover, the WTP for the enriched compote increased as more information on the positive properties of Aloe vera was given. Both health related and environment sustainability related messages increased the willingness to pay for the aloe vera enriched fruit jam | | | | | |
| Cattaneo et al. [125] | 2019 | Italy | 273 (M 46.1%; F 53.9%)<br><br>Age range: 18-72 years | Quantitative<br><br>(Interviewer-administered questionnaire) | Food Technology Neophobia Scale (FTNS); Consumers' attitude | Bread, tomato puree, apple puree; cheese and yogurt enriched with upcycled ingredients |
| | **Main outcomes**<br>Acceptability for foods containing food by-products as novel ingredients enriching food formulation resulted positively correlated with high level of education, as, in general, higher education levels also reduce consumers' neophobia. A significant effect of the main factors 'Food technology neophobia' and 'Information' on consumers' attitude towards food by-products reutilisation was found. The level of information provided, and the education level influenced consumers' attitude towards reuses of food by-products. Neophobia in relation to food technology was slightly but significantly influenced by education level | | | | | |
| Chezan et al. [119] | 2022 | United Kingdom, Germany, Romania, France, Denmark, Czech Republic, Hungary, Ireland, and Italy | 102; (M 20.6%, F 79.4%)<br>Age range: older than 18 years | Quantitative (Semi-structured interviews) | Food Technology Neophobia Score (FTNS); Perceived benefit (PB); Purchase intentions (PI); Definition of consumers groups (vegetarian; reducetarian; unrestricted; omnivore) | Meat substitutes enriched with fungal proteins |
| | **Main outcomes**<br>Fungal proteins were perceived as more beneficial from a societal perspective than a personal one. FTN was a significant but weak predictor PB and PI. PBs of fungal proteins were significantly higher among younger participants. People living in urban areas had a statistically significantly higher PI score than those living in rural areas. Familiarity with fungal protein, with other products containing mould and knowledge about mould and/or fungi and their association with familiar foods positively affected the acceptance of fungal novel food. Sensory attributes (similarity to meat - texture - taste - smell) and absence of additives and preservatives were the most important factors in the acceptance and success over time of meat substitutes. Price of meat represents a trigger price of what consumers are prepared to pay for its alternatives. Vegetarian consumers had lower levels of PI for fungal protein | | | | | |
| Coderoni and Perito [139] | 2020 | Italy | 477 (M 35.0%, F 65.0%)<br>Age range: older than 18 years | Quantitative (web-based questionnaire using scales) | Willingness to buy (WTB), ood neophobia (FN), food echnology neophobia (FTN) | Waste-to-value (WTV) foods enriched by olive leaves extracts, (salted "taralli", crackers and breadsticks) and mayonnaise |

| | | | | | | |
|---|---|---|---|---|---|---|
| | **Main outcomes**<br>FN and FTN resulted important factors affecting the acceptance of WTV food, and they are influenced by socio-demographic characteristics of respondents. Consumers accustomed to read food labels and trusting in environmental, or health benefits of these products are more likely to state a positive purchase intention. Generalised trust influenced the final choice when it interacts with some other personal traits: respondents who are older and have a lower income level, when showing higher generalised trust, are more likely to buy WTV food, while the opposite occurs for male and graduate respondents | | | | | |
| Coderoni and Perito [130] | 2021 | Italy | 317; (M 36.0%, F 64.0%)<br>Age range: 19-40 years | Quantitative: web-based questionnaire using scales | Villingness to buy (WTB), ood neophobia (FN), food echnology neophobia (FTN), nvironmental and health enefits perception | Waste-to-value (WTV) foods enriched by olive leaves extracts, (salted "taralli", crackers and breadsticks) and mayonnaise |
| | **Main outcomes**<br>Positive WTB is related to environmental, or health benefits attributed to upcycled foods. Gender, education level and income have a significant impact on WTB: being female negatively impact on the WTB; high education level and low income positively impact on WTB food produced with upcycled ingredients, FN and FTN negatively impacted on WTB food produced with upcycled ingredients, reading the food label can increase WTB. | | | | | |
| Coulthard et al. [133] | 2022 | United Kingdom | Study 1: 534; (M 29.0%, F 71.0%)<br>Age range: 18-72 years<br>Study 2: 160; (M 20.0%, F 80.0%)<br>Mean Age: 20.47 years | Quantitative: web-based questionnaire using scales | ood neophobia (FN), Adult/Adolescent Sensory Profile (AASP) Disgust Propensity and Sensitivity Scale-Revised (DPSS-R); Hospital Anxiety and Depression Scale (HADS) | Novel fruits and vegetables (karela, gourd, plantain, okra, cassava, custard fruit, guava, sharon fruit) |
| | **Main outcomes**<br>FN was positively associated with tactile and taste/smell sensitivity, anxiety, and disgust sensitivity. High disgust sensitivity, low perceived familiarity, and low associating to liked foods were associated with lower expected liking of the novel food images. Perception of a novel food as familiar and the expectation that it will taste like a previously liked familiar food, led to positive expectations of eating the food | | | | | |
| De Marchi et al. [134] | 2019 | Italy | 582; (M 49.0%, F 51.0%)<br>Age range: older than 18 years | Choice experiment Face-to-face interviews | Choosing option and preferences | CIS-genic apples |
| | **Main outcomes**<br>On overall respondents preferred conventional over CIS-genic apples, attributing them higher naturalness. Price is a determinant decreasing respondents' utility. For "Attentive" consumers technology of production mostly influenced the preferences, followed by the origin and the low price. "Technology indifferent" (ignoring the production technology) consumers were more influenced by price, environmental impact and Brand; "technology driven" consumers preferred conventional apples over CIS-genic ones | | | | | |
| Grasso and Asioli [126] | 2020 | United Kingdom | 106 (M 50.0%, F 50.0%)<br>Age range: 18-75 years | Qualitative (Online surveys and questionnaire) | Preference | Biscuits with upcycled ingredients |

| | | | | | | |
|---|---|---|---|---|---|---|
| | **Main outcomes**<br>Results showed heterogeneity in consumers' valuation. Most consumers had not heard of upcycled ingredients before, but they would consider buying foods with upcycled ingredients. A first group with "price sensitive" consumers expressed the strongest preferences for low price biscuits, a second group with "traditionalist" consumers showed rejection for upcycled sunflower flour, a third group, defined of "environmentalist" consumers, expressed the strongest preference for biscuits with the Carbon Trust label | | | | | |
| Niimi et al. [128] | 2022 | Sweden | Consumer test: 101 (M 42.6%, F 57.4%) Age range: older than 18 years<br>Online survey:288(M 24.2%, F 74.7%) Age range: older than 18 years | Online surveys and consumer test | Cooking ability (CAFPAS), Food neophobia (FN) | 6 Bolognese sauce formulation with beef (1 formulation), soybean-based (3), mycoprotein (1), and oat (1) |
| | **Main outcomes**<br>FN resulted negatively related to CAFPAS. Regarding to the overall liking, the only significant effect was of FNS. Liking of appearance of Soy_O formulation was liked significantly more, and that of Oats was liked significantly less, than all other samples. FNS was also a significant covariate for liking of appearance. Liking of aroma was lower for where Oats and Soy_H. Liking of taste/flavour of Oats was lower than all the other samples, and Soy_O was liked more than Soy_H, Soy_A, and Mycoprotein. The Bolognese sauces produced with the three soy products were relatively similar sensorially. For appearance, the presence of vegetables and red colour showed significant increases in liking. Attributes that significantly reduced liking for flavour taste were carboard and bitterness | | | | | |
| Noguerol et al. [129] | 2021 | Spain | 101 (M 32.7%, F 63.7%)<br>Age range: 18-64 years | Qualitative (questionnaires Projective mapping, Multifactor Analysis (MFA) | General nutrition knowledge, Motives underlying food choice, Health and ecological concern | Plant based sausages, burgers, vegetable steaks, salami, croquettes, meatballs, quinoa spread, sobrassada, pizza and quinoa with vegetables |
| | **Main outcomes**<br>Consumer categorisation showed a clear distinction into three groups of products. The first group contains the four labels of the two plant-based products which are breaded (croquettes and meatballs), without differentiating if they were original or clean label (CL). The second group collected both options of pizza and quinoa with vegetables, again without separating the original from the clean label. Finally, the third group contained the meat analogues like burgers, steaks and cold cuts, and the quinoa spreads, without separation of original and clean labels. Projective mapping showed that for consumers reducing or avoiding meat and other animal products the status of clean label is an important characteristic for their perception of plant-based foods, quite different to the map obtained for omnivorous consumers | | | | | |
| Pagliarini et al. [127] | 2021 | Italy | 302 (M 44.0%, F 56.0%)<br>Age range: 18-60 years | Quantitative (Labelled Affective magnitudo Scale) | Liking; familiarity; | Beetroot puree enriched with winemaking by-products |

| | | | | | | |
|---|---|---|---|---|---|---|
| | **Main outcomes** Phenols from winemaking by-products addition increased sourness, but did not affect hedonic ratings. A significant age effect was found on all sensory attributes perception. Sweet taste was rated higher by the youngest subjects, compared to older. Sourness, astringency and overall flavour were rated lower by the group with 31–45 years, comparatively to the groups from the other age ranges. A significant gender effect was found only on astringency perception, with men showing higher scores compared to women. One cluster of consumers preferred high phenol rich purees, with more intense sensory attributes and showed lower food neophobia and higher scores of emotional eating. One cluster gave higher liking scores to the samples without or with the lowest concentration of phenols from unripe grapes. No differences according to age and gender distributions have been highlighted in the two clusters | | | | | |
| Profeta et al. [117] | 2021 | Germany | 500(M 44.0%, F 56.0%) Age range:>18 years | Quantitative (online survey) | requency of consumption of: rganic free range meat, meat ubstitutes, meat replacers, veggie burgers). Preference or meat hybrid (60% meat nd 40% plant-based protein) nd for buying location | Meat hybrids |
| | **Main outcomes** More than half of consumers declared to substitute meat, at least occasionally. About half of the respondents declared sporadic responsible behaviour in terms of sustainability and health. However, most of the consumed meat alternatives have an animal origin (dairy products, fish, eggs). The findings of this study demonstrate that at least a substantial number of consumers is open-minded to the "meat hybrid" concept. Even a higher share believes that this new alternative is healthier, better for the environment and the animals in comparison to meat. Thus, there is chance that hybrids could serve as a low threshold option for a transition in the direction towards a more sustainable diet | | | | | |
| Proserpio et al. [131] | 2019 | Italy | 103 (M 55.0%, F 46.0%) Age range: 9-11 years | Quantitative (hedonic scales) | iking | Breadsticks added with mushroom (*P. Ostreatus*) at different concentration (0, 2, 4, 6%) |
| | **Main outcomes** Control samples (no addition) and low enriched obtained comparable scores. Children equally liked these samples, which were significantly preferred to samples with increasing added concentrations of P. ostreatus powder. Samples enriched with mushroom powder at 2% and 4% obtained similar liking scores to each other while the sample with the highest concentration of P. ostreatus powder received the lowest liking scores. However, all the vitamin D2-enriched samples were well accepted by the children. The main factor "gender" and the interaction "sample*gender" were not significant. No differences were found in sample liking scores in the low food neophobic group. Neophobic children gave the lowest liking rating to the sample with the highest concentration of mushroom sample. A similar trend was highlighted for the medium neophobic children | | | | | |
| Proserpio et al. [132] | 2019 | Italy | 202 (M 50.5%, F 49.5%) Age range: 13-18 years | Quantitative, hedonic scales | iking, food technology ieophobia, healthy eating core | Flat bread added with mushroom (*P. Ostreatus*) |

| | | | | | | |
|---|---|---|---|---|---|---|
| | **Main outcomes** The sample with mushroom powder added was generally well accepted, but hedonic responses among adolescents were different according to their food technology neophobia level and their food habits healthiness. Adolescents with a low food technology neophobia level and healthy eating behavior mostly appreciated the sample with mushroom powder added. People with neophobic and unhealthy eating behavior gave comparable hedonic scores to the two samples. A negative correlation was found between food technology neophobia level and healthy food habits | | | | | |
| Rocha et al. [121] | 2021 | Portugal | 60 (M 47.5%, F 52.5%) Age range: 8-12 years | Quantitative (focus group, hedonic scales) | Liking, sensory profile Check-All-That-Apply (CATA) | Sweet and salty biscuits with fermented grass pea flour at different concentration (from 0 to 40%) |
| | **Main outcomes** Children were able to discriminate the different samples with the hedonic scale and according to their sensory profile. The focus-group resulted an adequate tool to define CATA ballots and the CATA approach was suitable to assess how children discriminate the sensory profile of food products. The food neophobia level of the children negatively influenced the food acceptability | | | | | |
| Śmiglak-Krajewska et al. [123] | 2020 | Poland | 1067 (M 48.0%, F 52.0%) Age range: ≥18 years | Quantitative, online survey | ood choice motives | Legumes |
| | **Main outcomes** Legume products, generally acknowledged as animal protein alternative, in Poland are still scarcely consumed, although their popularity and appreciation by consumers are growing. Differences were observed in consumers knowledge and preferences according to their residential area: rural residents ranked their preferences for legumes as follow: peas, beans, lentils and soybeans; for residents in small cities the order of preference was beans, soybeans, chickpeas and broad beans; for residents of cities of more than 30,000 inhabitants, the ranking was: lentils, peas, soybeans and beans. These insights were related to the differences in diffusion of food shops to market among cities and rural areas | | | | | |
| Śmiglak-Krajewska & Wojciechowska-Solis [122] | 2021 | Poland | 1067 (M 48.0%, F 52.0%) Age range: ≥18 years | Quantitative, online survey | Frequency of pulse as meat substitute consumption | Soy milk, grains, tofu cottage cheese, half-products, e.g., soy chops, Products containing peas—and beans |
| | **Main outcomes** Consumption of pulses by Polish citizens resulted still low. Peas and beans resulted the most known and consumed. Results showed a strong influence of foreign cuisines, mainly Middle Eastern and Mediterranean, on Polish consumers' preference, Citizen not following any dietary regimen were less prone to consume high-protein plant products. People having specific dietary habits due to health or ethical reasons declared to consume pulses 1–2 a week to 3–4 times a week. Soya and lentils resulted more consumed by consumers limiting animal product in their diet or submitted to specific medical restrictions. On overall, scarce differences were observed between men and women but women paid more attention to the healthiness and nutritional value of pulses. Product taste and texture, scarce cooking skills and high content of carbohydrates of pulses resulted significant hindrances for a wider diffusion and consumption | | | | | |

| | | | | | | |
|---|---|---|---|---|---|---|
| Van 't Riet et al. [120] | 2016 | Greece; The Netherland; Spain and Poland | 1652 (M 52.2%, F 47.8%) Age range: 19-100 years | Quantitative: Choice experiment | Choice between alternative products; willingness to pay; | Novel fruits (genetically modified apples, cholesterol-lowering peaches, dried black currant with probiotic nutrients, and cholesterol-lowering orange juice |
| | **Main outcomes** Marketing claims and price information significantly affected consumers' choices. Naturalness of a product and low price positively influenced choices more than information on technological aspects. Country of consumers and product type influenced the effects of market claims and price. Time-until-expiration, were perceived in different way in different countries | | | | | |
| Yang et al. [124] | 2020 | United Kingdom | 100 (M 34.0%, F 76.0%) Age range: 18-74 years | Quantitative, hedonic scales, questionnaires | Overall liking, emotions, food neophobia | Biscuits and crackers added with Bambara groundnut (legume). |
| | **Main outcomes** No differences in liking resulted between standard and Bambara added products, under blind condition. Information about quality and sustainability enhanced acceptability of novel products containing Bambara flour. Despite a general tendency to rejection by neo-phobic consumers, food neophobia slightly influenced acceptability and emotional response towards Bambara added and standard products | | | | | |
